# Supplementary material for: CARINH, an Interferon-Induced LncRNA in Cancer and Inflammation
Source: Noncoding RNA. 2025 Nov 21;11(6):79. doi: 10.3390/ncrna11060079 (PMC12642018; doi:10.3390/ncrna11060079)
Supplement: Supplementary file 1 [file ncrna-11-00079-s001.zip › ncrna-3946111-supplementary.pdf]

Supplementary Table S1. Predicted miRNA interactions with *CARINH* variants

ENST00000612967.2

Target Rank	Target Score	miRNA Name
1	86	hsa-miR-4474-3p
2	83	hsa-miR-6792-3p
3	83	hsa-miR-4691-5p
4	82	hsa-miR-423-5p
5	82	hsa-miR-3184-5p
6	81	hsa-miR-5787
7	81	hsa-miR-4505
8	80	hsa-miR-661
9	77	hsa-miR-610
10	77	hsa-miR-4791
11	77	hsa-miR-4443
12	76	hsa-miR-9851-5p
13	72	hsa-miR-3201
14	71	hsa-miR-7107-3p
15	71	hsa-miR-6753-3p
16	69	hsa-miR-511-3p
17	66	hsa-miR-4758-3p
18	65	hsa-miR-345-5p
19	65	hsa-miR-4516
20	64	hsa-miR-4319
21	63	hsa-miR-500b-5p
22	63	hsa-miR-362-5p
23	63	hsa-miR-29b-1-5p
24	63	hsa-miR-6722-3p
25	62	hsa-miR-4685-5p
26	62	hsa-miR-1909-3p
27	62	hsa-miR-6837-5p
28	61	hsa-miR-4430
29	61	hsa-miR-3652
30	60	hsa-miR-4329
31	60	hsa-miR-4668-5p
32	60	hsa-miR-5197-3p
33	59	hsa-miR-1321
34	59	hsa-miR-4650-5p
35	59	hsa-miR-939-3p
36	59	hsa-miR-3689f

37	59	hsa-miR-3689e
38	59	hsa-miR-3689b-5p
39	59	hsa-miR-3689a-5p
40	59	hsa-miR-431-5p
41	58	hsa-miR-1915-3p
42	58	hsa-miR-4688
43	58	hsa-miR-6764-5p
44	58	hsa-miR-6798-5p
45	58	hsa-miR-7113-3p
46	57	hsa-miR-10397-5p
47	56	hsa-miR-612
48	55	hsa-miR-10226
49	55	hsa-miR-194-5p
50	55	hsa-miR-4660
51	55	hsa-miR-1913
52	54	hsa-miR-6842-3p
53	54	hsa-miR-432-5p
54	54	hsa-miR-6752-3p
55	53	hsa-miR-6743-5p
56	53	hsa-miR-4739
57	53	hsa-miR-7976
58	52	hsa-miR-4756-5p
59	52	hsa-miR-6775-3p
60	52	hsa-miR-1286
61	52	hsa-miR-450a-2-3p
62	52	hsa-miR-646
63	52	hsa-miR-4447
64	51	hsa-miR-6748-5p
65	50	hsa-miR-644a
66	50	hsa-miR-3187-5p
67	50	hsa-miR-6857-5p
68	50	hsa-miR-5703
69	50	hsa-miR-4530
70	50	hsa-miR-4434

ENST00000337752.6

Target Rank	Target Score	miRNA Name
1	92	hsa-miR-4516
2	89	hsa-miR-3194-3p
3	89	hsa-miR-8485

4	88	hsa-miR-6792-3p
5	87	hsa-miR-10401-5p
6	87	hsa-miR-6773-5p
7	86	hsa-miR-4691-5p
8	86	hsa-miR-8080
9	86	hsa-miR-5703
10	86	hsa-miR-4434
11	85	hsa-miR-6724-5p
12	85	hsa-miR-4443
13	84	hsa-miR-4278
14	83	hsa-miR-6867-5p
15	83	hsa-miR-5196-5p
16	83	hsa-miR-4747-5p
17	82	hsa-miR-384
18	78	hsa-miR-4430
19	78	hsa-miR-3652
20	78	hsa-miR-6513-3p
21	77	hsa-miR-4534
22	77	hsa-miR-1305
23	76	hsa-miR-411-5p
24	75	hsa-miR-6732-5p
25	74	hsa-miR-6769b-3p
26	74	hsa-miR-4723-3p
27	74	hsa-miR-8082
28	73	hsa-miR-3183
29	73	hsa-miR-552-5p
30	72	hsa-miR-146a-3p
31	72	hsa-miR-5197-5p
32	72	hsa-miR-578
33	72	hsa-miR-6510-5p
34	71	hsa-miR-4738-3p
35	71	hsa-miR-4632-5p
36	71	hsa-miR-20a-3p
37	71	hsa-miR-6892-3p
38	70	hsa-miR-552-3p
39	70	hsa-miR-4744
40	70	hsa-miR-7856-5p
41	69	hsa-miR-6819-5p
42	69	hsa-miR-608
43	69	hsa-miR-4651
44	69	hsa-miR-4755-3p

45	69	hsa-miR-4654
46	69	hsa-miR-7113-3p
47	68	hsa-miR-3120-3p
48	68	hsa-miR-6868-3p
49	67	hsa-miR-3688-5p
50	67	hsa-miR-145-5p
51	67	hsa-miR-6879-5p
52	67	hsa-miR-6735-5p
53	67	hsa-miR-4436b-3p
54	67	hsa-miR-7843-5p
55	67	hsa-miR-4769-5p
56	67	hsa-miR-33a-3p
57	66	hsa-miR-4447
58	66	hsa-miR-4677-5p
59	65	hsa-miR-6887-5p
60	65	hsa-miR-6795-5p
61	65	hsa-miR-5195-3p
62	65	hsa-miR-1252-3p
63	65	hsa-miR-6752-3p
64	64	hsa-miR-6739-3p
65	64	hsa-miR-493-5p
66	64	hsa-miR-217-3p
67	64	hsa-miR-6842-3p
68	64	hsa-miR-1207-5p
69	64	hsa-miR-7978
70	63	hsa-miR-320e
71	63	hsa-miR-4710
72	63	hsa-miR-218-5p
73	62	hsa-miR-4530
74	62	hsa-miR-5691
75	62	hsa-miR-4319
76	62	hsa-miR-125b-5p
77	62	hsa-miR-125a-5p
78	61	hsa-miR-650
79	61	hsa-miR-3612
80	61	hsa-miR-4302
81	60	hsa-miR-6737-5p
82	60	hsa-miR-6885-5p
83	60	hsa-miR-328-5p
84	60	hsa-miR-491-5p
85	60	hsa-miR-3124-3p

86	60	hsa-miR-642b-3p
87	60	hsa-miR-642a-3p
88	60	hsa-miR-4429
89	60	hsa-miR-320d
90	60	hsa-miR-320c
91	60	hsa-miR-320b
92	60	hsa-miR-320a-3p
93	60	hsa-miR-4662a-3p
94	59	hsa-miR-4656
95	59	hsa-miR-200b-5p
96	59	hsa-miR-200a-5p
97	59	hsa-miR-7111-3p
98	59	hsa-miR-16-1-3p
99	59	hsa-miR-5787
100	59	hsa-miR-3166
101	59	hsa-miR-4492
102	59	hsa-miR-4505
103	59	hsa-miR-4531
104	59	hsa-miR-5006-3p
105	59	hsa-miR-4755-5p
106	59	hsa-miR-520f-5p
107	59	hsa-miR-6805-3p
108	58	hsa-miR-5698
109	58	hsa-miR-4521
110	58	hsa-miR-125a-3p
111	58	hsa-miR-1827
112	58	hsa-miR-766-3p
113	58	hsa-miR-4481
114	57	hsa-miR-4441
115	57	hsa-miR-644a
116	57	hsa-miR-18a-3p
117	56	hsa-miR-6812-5p
118	56	hsa-miR-3646
119	56	hsa-miR-4717-3p
120	56	hsa-miR-3944-5p
121	56	hsa-miR-1250-3p
122	56	hsa-miR-7150
123	55	hsa-miR-940
124	55	hsa-miR-4664-5p
125	55	hsa-miR-4468
126	55	hsa-miR-623

127	55	hsa-miR-6743-5p
128	55	hsa-miR-4688
129	55	hsa-miR-6721-5p
130	55	hsa-miR-7109-5p
131	55	hsa-miR-6722-3p
132	55	hsa-miR-147a
133	55	hsa-miR-3180-5p
134	55	hsa-miR-3125
135	55	hsa-miR-7974
136	54	hsa-miR-30e-3p
137	54	hsa-miR-30d-3p
138	54	hsa-miR-30a-3p
139	54	hsa-miR-4535
140	54	hsa-miR-12115
141	53	hsa-miR-4763-3p
142	53	hsa-miR-4470
143	53	hsa-miR-7155-3p
144	53	hsa-miR-3136-3p
145	53	hsa-miR-4456
146	53	hsa-miR-7111-5p
147	53	hsa-miR-6870-5p
148	53	hsa-miR-4723-5p
149	53	hsa-miR-6780b-5p
150	53	hsa-miR-4725-3p
151	53	hsa-miR-4427
152	52	hsa-miR-1909-3p
153	52	hsa-miR-6865-3p
154	52	hsa-miR-6817-3p
155	52	hsa-miR-6074
156	52	hsa-miR-6893-5p
157	52	hsa-miR-6808-5p
158	51	hsa-miR-423-5p
159	51	hsa-miR-3184-5p
160	51	hsa-miR-22-5p
161	51	hsa-miR-4784
162	51	hsa-miR-3150b-3p
163	51	hsa-miR-574-5p
164	51	hsa-miR-1185-2-3p
165	51	hsa-miR-1185-1-3p
166	51	hsa-miR-342-5p
167	50	hsa-miR-6124

168	50	hsa-miR-3664-3p
169	50	hsa-miR-187-5p

ENST00000407797.6

Target Rank	Target Score	miRNA Name
1	90	hsa-miR-4687-3p
2	90	hsa-miR-4516
3	87	hsa-miR-12120
4	84	hsa-miR-6792-3p
5	84	hsa-miR-4691-5p
6	83	hsa-miR-7845-5p
7	81	hsa-miR-5703
8	81	hsa-miR-4434
9	81	hsa-miR-6727-5p
10	79	hsa-miR-6071
11	79	hsa-miR-5683
12	77	hsa-miR-1303
13	77	hsa-miR-6875-3p
14	71	hsa-miR-6783-3p
15	71	hsa-miR-4531
16	71	hsa-miR-1343-3p
17	67	hsa-miR-3120-5p
18	67	hsa-miR-3925-5p
19	65	hsa-miR-17-3p
20	65	hsa-miR-1910-5p
21	63	hsa-miR-766-3p
22	62	hsa-miR-3692-5p
23	62	hsa-miR-93-3p
24	60	hsa-miR-4664-5p
25	60	hsa-miR-19b-3p
26	60	hsa-miR-19a-3p
27	60	hsa-miR-1911-3p
28	60	hsa-miR-6817-5p
29	60	hsa-miR-4769-3p
30	60	hsa-miR-7855-5p
31	59	hsa-miR-890
32	57	hsa-miR-4673
33	57	hsa-miR-432-5p
34	57	hsa-miR-4530
35	55	hsa-miR-4726-5p

36	55	hsa-miR-4640-5p
37	53	hsa-miR-4319
38	53	hsa-miR-342-5p
39	53	hsa-miR-4645-5p
40	52	hsa-miR-5197-3p
41	52	hsa-miR-513b-3p
42	52	hsa-miR-670-5p
43	51	hsa-miR-4463
44	50	hsa-miR-527
45	50	hsa-miR-518a-5p
46	50	hsa-miR-1267
